# 反相/亲水相互作用双模式交替液相色谱-串联质谱分离系统的构建及其在代谢物全谱研究中的应用

**DOI:** 10.3724/SP.J.1123.2024.05010

**Published:** 2024-12-08

**Authors:** Yan HUANG, Xing ZHANG, Tingting JIANG, Yawen ZHOU, Peng JIANG, Hongfeng YIN, Tao TANG

**Affiliations:** 浙江福立分析仪器有限公司, 浙江 台州 317500; Zhejiang Fuli Analytical Instruments Co., Ltd., Taizhou 317500, China

**Keywords:** 反相液相色谱, 亲水相互作用液相色谱, 代谢物覆盖度, 代谢谱分析, 双模式分离, reversed-phase liquid chromatography, hydrophilic interaction liquid chromatography, metabolite coverage, metabolic profiling, dual-mode separation

## Abstract

生物样品通常具有基质复杂、种类繁多、理化性质差异极大等特征,采用一种分离模式通常只能得到样品的部分信息,发展多模式结合的分析方法,对于样品的全景特征分析、生物标志物发现皆具有重要意义。本文将HILIC和RPLC两根色谱柱与一台自动进样器、两个输液泵结合,并耦合到质谱仪上采集数据,构建了一种基于单进样器的HILIC/RPLC-MS/MS双模式交替分离系统。构建的系统通过一个两位六通阀和一个两位十通阀使自动进样器始终与质谱仪在同一流路上,但是与HILIC和RPLC两根色谱柱交替相连。当自动进样器与HILIC色谱柱相连时,HILIC运行梯度洗脱,RPLC处于平衡状态,反之亦然,使得总分析时间减少约40%,而且可避免数据集重叠。采用该系统对14种代表性的代谢物(包括有机酸、氨基酸、胆碱和溶血磷脂等)进行了分析,结果表明,相比于RPLC-MS/MS单模式系统和HILIC-MS/MS单模式系统,HILIC/RPLC-MS/MS双模式交替分离系统覆盖的代谢物数量更多。在尿液基质中,14种代谢物的定量线性关系均较好,相关系数(*R*^2^)大于0.99,方法的检出限和定量限分别为0.02~42.86 ng/mL和0.08~142.86 ng/mL,在低、中、高3个加标水平下的回收率为85.2%~113.9%,日内、日间精密度(RSD)小于10%。该系统仪器配置简单,操作便捷,实用性强,适合在临床上用于代谢物全谱研究,也可用于蛋白质组学、糖组学的研究,尤其适合大队列样本的组学分析,具有广阔的应用前景。

## Abstract (en)

As one of the youngest “omics” disciplines, metabolomics, more specifically, metabolite profiling, enables the comprehensive quantitative analysis of a primary metabolome. At present, comprehensive metabolite profiling analysis is a challenging task owing to the greatly different physicochemical properties of metabolites and the complexity of sample matrices, especially given the wide variety of biological samples. Consequently, the development of a multimodal separation method with higher metabolite coverage than single-mode chromatography is necessary for comprehensive metabolite profiling analysis and biomarker discovery.

Reversed-phase liquid chromatography (RPLC) and hydrophilic interaction liquid chromatography (HILIC) are used to analyze nonpolar and polar metabolites, respectively. HILIC and RPLC separation results are commonly combined to achieve comprehensive metabolite profiling analysis. However, this practice leads to overlapping datasets and extended analysis times. Alternatively, multiple LC systems can be coupled to generate combined HILIC and RPLC separation results. In this study, we developed an alternating HILIC/RPLC-tandem mass spectrometry (MS/MS) dual-mode separation system consisting of one autosampler, two high-pressure LC pumps, a HILIC column, an RP column, a 6-port 2-position switching valve, a 10-port 2-position switching valve, and a mass spectrometer for the comprehensive and high-throughput analysis of polar and nonpolar metabolites.

In this configuration, the autosampler is always on the same flow path as the mass spectrometer, whereas the HILIC and RP columns are switched in and out of the main flow path. At the initial stage of the analysis, the autosampler is connected to a HILIC column, followed by the mass spectrometer. While gradient elution is performed on the HILIC column, the RP column is equilibrated using the second high-pressure pump. After HILIC separation, the 6-port 2-position and 10-port 2-position switching valves are switched simultaneously. At this stage of the analysis, the autosampler is connected to the RP column, followed by the mass spectrometer, while the HILIC column is equilibrated. This process is repeated for the subsequent samples. Because the equilibration time of one column overlaps with the gradient elution of the other column, the total analysis time can be reduced by approximately 40%. This system was used to analyze 14 representative endogenous metabolites, including four common types: organic acids, amino acids, choline, and lysophospholipids. The alternating HILIC/RPLC-MS/MS dual-mode separation system had high metabolite coverage and could analyze all 14 representative metabolites simultaneously. By contrast, only 10 of the 14 metabolites could be analyzed using the HILIC-only system. The HILIC-only system failed to analyze organic acids. On the other hand, the RP-only system failed to analyze hydrophilic amino acids, choline, and lysophospholipids. These findings indicate that only 6 of the 14 representative metabolites could be analyzed by the RP-only system. Method validation was conducted to ensure the accuracy of the proposed method. Fourteen representative metabolites were spiked into a urine matrix for validation. All 14 metabolites exhibited linear responses, with correlation coefficients greater than 0.99. The limits of detection (LODs) were in the range of 0.02-42.86 ng/mL, and the limits of quantification (LOQs) were in the range of 0.08-142.86 ng/mL. The average recoveries at three spiked levels were in the range of 85.2%-113.9%. The intra- and inter-day precisions (RSDs) were less than 10%. The proposed instrument configuration and dual-mode method are practical and easy to use for metabolite profiling research in clinical applications such as disease diagnosis, drug development, and biomarker discovery. Furthermore, the proposed instrument configuration and method could potentially be used to analyze other complex samples in proteomics and glycomics research.

代谢组学是对生物体内所有小分子代谢物进行定性定量分析并寻找代谢物与生理病理变化相对关系的科学^[1,2]^。相比于其他组学,代谢组学反映生命体已经发生的生物学事件,能够更直接准确地反映生命体终端和表型信息^[3]^,在发病机理探索、生物标志物发现和药物开发等精准医学和转化医学中发挥着重要作用^[4⇓-6]^。反相液相色谱-质谱(RPLC-MS)方法因其卓越的分离性能和良好的分析重复性,已成为代谢组学研究中应用最为广泛的分析技术之一^[7⇓-9]^。但是,RPLC对强极性代谢物保留弱,致使大量强极性组分在死时间附近出峰,互相干扰,进而导致代谢物定性困难。亲水相互作用色谱(HILIC)作为RPLC的补充技术^[10,11]^,对大多数极性化合物有很好的保留和分离^[12,13]^。近年来,随着HILIC色谱柱性能的提高,HILIC-MS技术在代谢组学研究中的应用也越来越广泛^[14,15]^。

二维液相色谱是分离分析复杂体系样品的重要工具^[16]^,在代谢组学^[17]^、蛋白质组学^[18]^、糖组学^[19]^等领域有着广泛的应用。结合具有不同分离机理的色谱模式是二维液相色谱技术的优势之一,如将正相液相色谱(NPLC)或者HILIC和RPLC结合,提高分辨率^[20,21]^。Contrepois等^[22]^和张国瑗等^[23]^的研究表明,与只有一种分离模式相比,将HILIC和RPLC分离结合在一起可以显著扩大代谢物的覆盖度,但分析时间是两种分离模式分析时间的总和。Klavins等^[24]^建立了平行柱LC分析方法,用于定性定量分析中心碳通路中的代谢物,可将现有的RPLC和HILIC方法结合起来,缩短分析时间并提高通量,但在代谢物覆盖度方面并没有明显改善。Lv等^[25]^将双色谱系统与质谱联用,开发了一种RPLC和HILIC交替分析的方法,可以轮流进行梯度洗脱和色谱柱平衡,分析通量和覆盖度均显著提高,但需要多个液相色谱系统。Guo等^[26]^建立了在线二维液相色谱方法,用于同时分析各种生物样品的亲水和疏水代谢物。二维液相色谱具有更好的分离效果,同时获得更多的样品信息,然而传统二维液相色谱方法存在稀释效应严重、灵敏度低、数据处理困难、装置相对复杂、分析时间长等问题。

此外,代谢组学研究的一些分析需求并不需要非常全面地了解样品信息,只是对其中的具有不同物性特征的重要组分感兴趣,需要有相对简单的装置可以完成RPLC和HILIC的双模式分析。本研究采用一套常规液相色谱、一个输液泵和两个切换阀构建了一种基于单进样器的HILIC/RPLC-MS/MS双模式交替分离系统,只需一个自动进样器即可提高代谢物的覆盖度。HILIC和RPLC分离在一个自动进样器和两个输液泵上交替进行,它们与质谱仪联用,质谱仪连续获得HILIC和RPLC的梯度洗脱周期数据。当自动进样器与某根色谱柱串联,这根色谱柱就处于分离状态,另一根色谱柱则处于柱平衡状态。采用构建的系统对14种代表性的代谢物(包括4种有机酸、7种氨基酸、胆碱和2种溶血磷脂)进行了分析,结果表明,该系统的代谢物覆盖度高于任何单模式系统的代谢物覆盖度,且分析时间低于两个单模式系统分析时间之和。该系统仪器配置简单,代谢物覆盖度高,可以在设定的批处理模式下运行,自动化程度高,非常适用于临床样品的代谢谱学研究,也可以将该系统用于蛋白质组学、糖组学的研究。

## 1 实验部分

### 1.1 仪器、试剂与材料

LC5190高效液相色谱仪,配有1个自动进样器、2个二元泵、1个柱温箱(内置1个两位六通阀和1个两位十通阀)、LC5s色谱工作站(浙江福立分析仪器有限公司); Triple Quad^TM^ 4500质谱仪(美国AB Sciex公司); HC-3018R高速冷冻离心机(安徽中科中佳科学仪器有限公司); DLAB MX-S型可调涡旋混匀仪(北京大龙兴创实验仪器股份公司);所有实验用水由Milli-Q超纯水系统(美国Millipore公司)制得。

甲醇、乙腈、异丙醇(色谱纯,美国TEDIA公司);甲酸、乙酸铵(色谱纯,德国Merck公司);人工尿液(上海源叶生物科技有限公司); 1-十六烷酰基-*sn*-甘油-3-磷酸乙醇胺(16∶0 Lyso PE)、1-十八酰基-*sn*-甘油-3-磷酰胆碱(18∶0 Lyso PC)标准品(德国Merck公司),纯度>99%; *α*-酮戊二酸、富马酸、顺式-乌头酸、琥珀酸、肌氨酸、L-丙氨酸、L-组氨酸、赖氨酸、L-谷氨酸、L-亮氨酸、L-苯丙氨酸、胆碱等12个标准品均购自上海安谱实验科技有限公司,纯度均大于98%;内标氯化胆碱-d4(德国Merck公司),纯度>98%。

### 1.2 实验条件

#### 1.2.1 标准溶液的配制

分别称取一定量的标准品置于5 mL棕色容量瓶中,用纯水溶解(其中L-谷氨酸、18∶0 Lyso PC和16∶0 Lyso PE分别用0.1%甲酸水溶液、甲醇和甲醇-三氯甲烷(1∶1, v/v)溶解),配制成1 mg/mL的标准储备液,于-20 ℃冰箱保存。

采用HILIC/RPLC-MS/MS双模式方法分析时,5种亲水氨基酸(肌氨酸、L-丙氨酸、L-组氨酸、赖氨酸、L-谷氨酸)、胆碱和2种溶血磷脂(18∶0 Lyso PC和16∶0 Lyso PE)按照一定比例混合,并用异丙醇-乙腈-人工尿液(2∶2∶1, v/v/v)稀释成混合标准工作液;4种有机酸(*α*-酮戊二酸、富马酸、顺式-乌头酸、琥珀酸)和2种非极性氨基酸(L-亮氨酸、L-苯丙氨酸)按照一定比例混合,并用甲醇-人工尿液(1∶1, v/v)稀释成混合标准工作液。

采用RPLC-MS/MS单模式方法分析时,所有标准储备液按照一定比例混合,并用甲醇-人工尿液(1∶1, v/v)稀释成混合标准工作液;采用HILIC-MS/MS单模式方法分析时,所有标准储备液按照一定比例混合,并用异丙醇-乙腈-人工尿液(2∶2∶1, v/v/v)稀释成混合标准工作液。

#### 1.2.2 样品前处理

从-80 ℃冰箱中取出人工尿液样品,置于4 ℃解冻。取200 μL样品至2 mL离心管中,向其中加入800 μL含有内标(125 μg/mL氯化胆碱-d4)的异丙醇-乙腈(1∶1, v/v)并涡旋混匀1 min后,在15200 r/min、4 ℃条件下离心20 min,取上清液过0.22 μm尼龙滤膜用于HILIC分析。另取500 μL样品至2 mL离心管中,向其中加入500 μL甲醇并涡旋混匀1 min后,在15200 r/min、4 ℃条件下离心20 min,取上清液过0.22 μm尼龙滤膜用于RPLC分析。

### 1.3 仪器条件

#### 1.3.1 HILIC/RPLC双模式色谱条件

HILIC模式:采用ChromCore HILIC-ZW色谱柱(150 mm×3.0 mm, 3 μm),流动相A为乙腈,流动相B为50 mmol/L乙酸铵水溶液(甲酸调pH=3.0),梯度洗脱(0~1 min, 10%B; 1~10 min, 10%B~50%B; 10~15 min, 50%B),色谱柱平衡(0~10 min, 10%B),流速为0.4 mL/min,色谱柱柱温为30 ℃,进样体积为5 μL。

RPLC模式:采用ChromCore AR C18色谱柱(150 mm×3.0 mm, 3 μm),流动相A为甲醇,流动相B为0.1%甲酸水溶液,色谱柱平衡和梯度洗脱(0~17 min, 95%B; 17~19 min, 95%B~60%B; 19~24 min, 60%B~0B; 24~26 min, 0B),流速为0.4 mL/min,色谱柱柱温为30 ℃,进样体积为2 μL。

切换阀:阀1, 0 min (1-6), 15 min (1-2), 26 min (1-6);阀2, 0 min (1-10), 15 min (1-2); 26 min (1-10)(见图1)。

**图 1:**
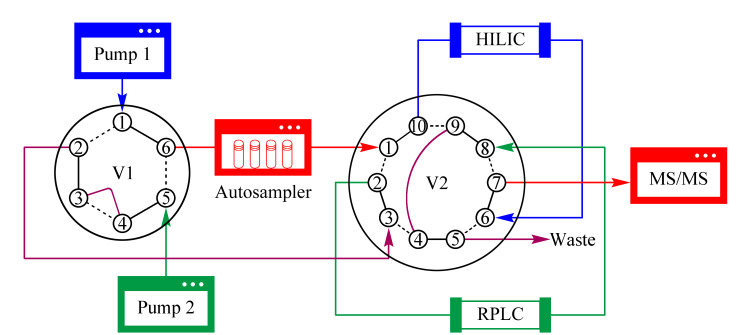
单进样器HILIC/RPLC-MS/MS双模式交替分离系统的流路示意图

#### 1.3.2 RPLC单模式色谱条件

为进一步验证所建立的HILIC/RPLC-MS/MS双模式交替分离系统在代谢物覆盖度方面的优势,我们同时使用RPLC-MS/MS单模式方法进行代谢物分析。通过条件优化,采用ChromCore AR C18色谱柱(150 mm×3.0 mm, 3 μm),流动相A为甲醇,流动相B为0.1%甲酸水溶液,梯度洗脱(0~1 min, 95%B; 1~3 min, 95%B~60%B; 3~8 min, 60%B~0B, 8~10 min, 0B; 10~10.1 min, 0B~95%B; 10.1~16 min, 95%B),流速为0.4 mL/min,色谱柱柱温为30 ℃;进样体积为2 μL。

#### 1.3.3 HILIC单模式色谱条件

同样作为对比,我们使用HILIC-MS/MS单模式方法进行代谢物分析。通过条件优化,采用ChromCore HILIC-ZW色谱柱(150 mm×3.0 mm, 3 μm),流动相A为乙腈,流动相B为50 mmol/L乙酸铵水溶液(甲酸调pH=3.0),梯度洗脱(0~1 min, 10%B; 1~10 min, 10%B~50%B; 10~15 min, 50%B; 15~15.1 min, 50%B~10%B; 15.1~25 min, 10%B),流速为0.4 mL/min,色谱柱柱温为30 ℃;进样体积为5 μL。

#### 1.3.4 质谱条件

离子源:电喷雾离子源(ESI),正、负离子模式;扫描模式:多反应监测模式(MRM);电喷雾电压:5500 V(正离子)、-4500 V(负离子);离子源温度:500 ℃;气帘气压力:241 kPa;雾化气压力:345 kPa;辅助气压力:379 kPa。14种代谢物和内标的质谱参数见表1。

**表 1:** 14种代谢物和内标的质谱参数

No.	Compound	Parent ion (m/z)	Daughter ion (m/z)	Declustering potential/ V	Collision energy/ V
1	sarcosine	90.1	44.2^*^	20	16
			72.1	20	16
2	L-alanine	90.1	44.2^*^	20	16
			72.1	20	16
3	L-histidine	156.0	110.0^*^	50	19
			138.1	50	10
4	lysine	147.1	84.0^*^	40	20
			130.1	40	15
5	L-glutamic	148.0	84.0^*^	40	20
	acid		130.0	40	15
6	choline	104.0	60.0^*^	60	23
			45.1	60	31
7	18∶0 Lyso PC	524.4	184.0^*^	120	38
			104.1	120	38
8	16∶0 Lyso PE	454.3	313.0^*^	38	29
			116.0	38	29
9	L-leucine	132.0	85.9^*^	40	15
			90.0	40	14
10	L-phenylalanine	166.1	120.0^*^	50	18
			103.0	50	38
11	α-ketoglutaric	144.9	100.9^*^	-27	-11
	acid		57.0	-27	-16
12	fumaric acid	115.0	71.0^*^	-60	-11
			44.9	-50	-30
13	cis-aconitic	172.9	84.9^*^	-20	-17
	acid		111.0	-20	-12
14	succinic acid	117.0	73.0^*^	-25	-15
			99.0	-25	-14
15	choline-d4	108.0	58.0^*^	60	35
			60.0	60	35

* Quantitative ion. 18∶0 Lyso PC: 1-stearoyl-2-hydroxy-*sn*-glycero-3-phosphocholine; 16∶0 Lyso PE: 1-palmitoyl-2-hydroxy-*sn*-glycero-3-phosphoethanolamine.

## 2 结果与讨论

### 2.1 单进样器HILIC/RPLC-MS/MS双模式交替分离系统的构建

色谱分离过程通常包括梯度洗脱和柱平衡两个阶段,梯度洗脱可以获取有效的色谱图数据,柱平衡则是获得良好系统重复性的必要保证。为了节省分析时间和提高通量,同时最大程度简化系统,构建了单进样器HILIC/RPLC-MS/MS双模式交替分离系统,通过同时切换两位六通阀和两位十通阀,使自动进样器始终与MS/MS相连,连续获取HILIC和RPLC梯度洗脱周期的数据。

系统流路如图1所示。泵1和泵2分别输送HILIC和RPLC梯度洗脱和柱平衡流动相,初始条件下,阀1和阀2均处于实线位置,此时泵1与自动进样器、质谱相连,HILIC进行梯度洗脱、质谱分析,泵2进行RPLC柱平衡,HILIC梯度洗脱完成后,同时切换阀1和阀2均至虚线位置,使泵2与自动进样器、质谱相连,进行RPLC梯度洗脱、质谱分析,泵1对HILIC柱进行平衡,如此反复进行,质谱只获取HILIC和RPLC梯度洗脱周期的数据,解决了色谱柱平衡时间长的问题,分析通量大大提高。

系统采样如图2所示。首先测量自动进样器的进样时间,大约需要1.0 min。由于该系统配置一个自动进样器,进样触发均由HILIC序列执行,HILIC序列提交后触发RPLC运行,RPLC运行后触发质谱采集数据,因此将HILIC序列运行分为HILIC有效梯度洗脱序列运行(对应于HILIC的有效梯度洗脱时间,15 min)和HILIC柱平衡序列运行(对应于HILIC的柱平衡时间,10 min), RPLC运行时间为26 min(对应于RPLC的柱平衡时间15 min、进样时间1 min和有效梯度洗脱时间10 min)。提交HILIC有效梯度洗脱序列后,HILIC进样分析,RPLC运行进行柱平衡,质谱采集HILIC数据,HILIC有效梯度洗脱序列结束后,同时切换阀1和阀2,使自动进样器进入RPLC流路,同时提交HILIC柱平衡序列,RPLC进样分析,运行梯度洗脱,HILIC进行柱平衡,质谱采集RPLC数据,直至HILIC柱平衡序列结束。该系统以质谱连续采集一组HILIC和RPLC的梯度洗脱数据为一个分析周期,质谱采集时间为26 min,因此总分析时间为26 min,而两次独立分析需要41 min,因此节省了大约40%的分析时间。同时,HILIC和RPLC色谱柱得到了充分的再平衡,保证了方法的重复性和可靠性。

**图 2:**
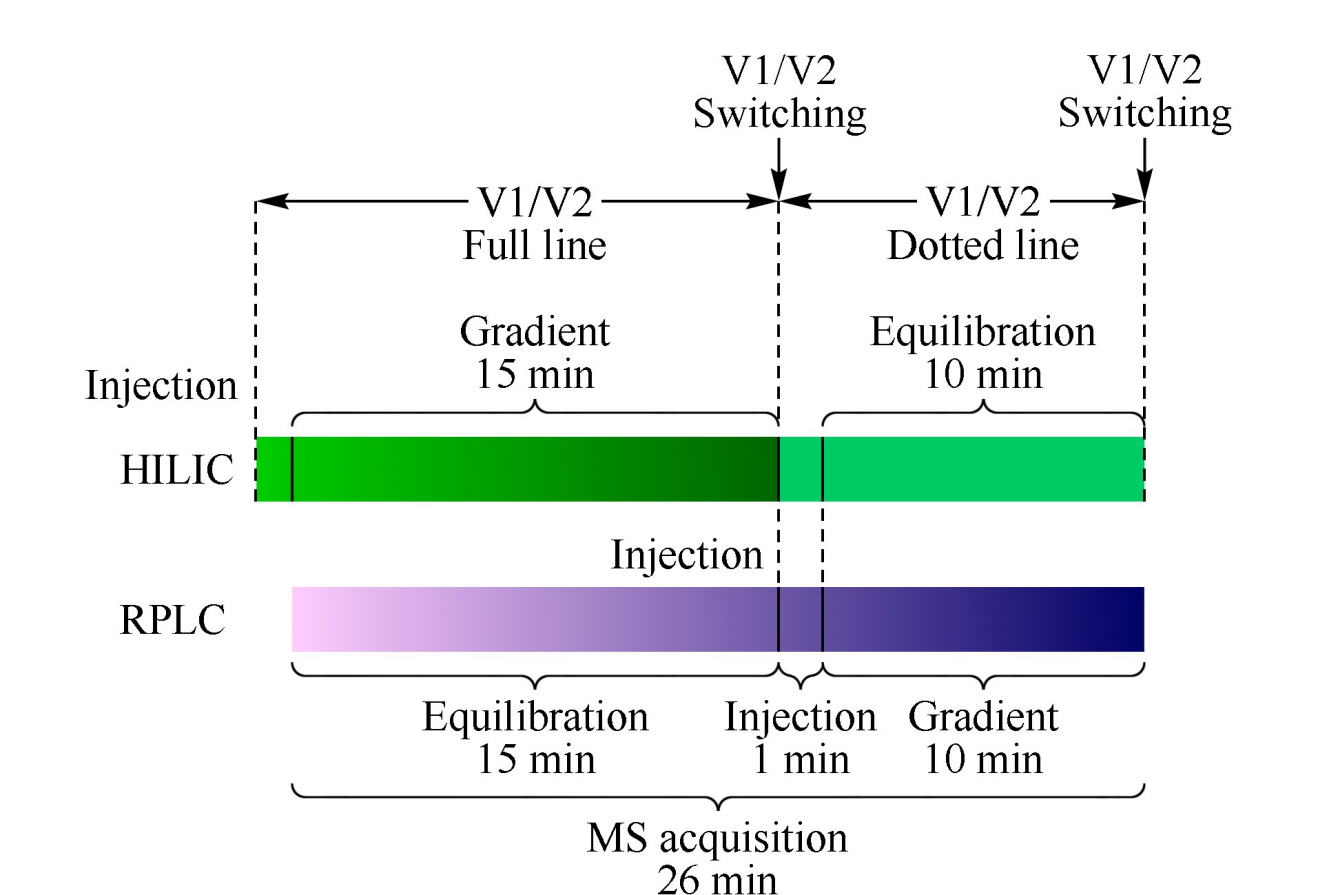
单进样器HILIC/RPLC-MS/MS双模式交替分离系统采样示意图

### 2.2 色谱条件优化

对于RPLC方法优化,为了提高极性代谢物的保留,采用了与100%含水流动相兼容的固定相(ChromCore AR C18色谱柱)为RPLC分离柱。对于HILIC方法优化,由于HILIC基于多模式保留机制,开发HILIC方法的难度比开发RPLC方法更大,因此,本研究着重对HILIC方法进行了优化。

首先是HILIC色谱柱的筛选,考察了ChromCore HILIC-Amide柱(150 mm×3.0 mm, 3 μm)和ChromCore HILIC-ZW柱(150 mm×3.0 mm, 3 μm)对峰形和分离效果的影响。结果表明,两者对待测物的保留性能一致,但是ChromCore HILIC-ZW柱对氨基酸,尤其是L-组氨酸和赖氨酸等碱性更强的化合物表现出更好的峰形,因此选择ChromCore HILIC-ZW柱(150 mm×3.0 mm, 3 μm)柱为HILIC分离柱。

其次是HILIC流动相的选择,由于ChromCore HILIC-ZW固定相基于全多孔硅胶颗粒,在高pH条件下不稳定,只能在低pH条件下使用,因此选择流动相pH为3.0。肌氨酸和L-丙氨酸为同分异构体,必须实现基线分离才能进行准确定量,通过调整梯度的斜率,将梯度变得更平缓,可以延长分析物与固定相的相互作用时间,有望改善分离效果,本研究对比了水相在5、9和12 min内从10%升到50%时的分离情况。结果表明,水相在9 min内从10%升到50%,肌氨酸和L-丙氨酸可以达到基线分离。适当调节流动相的缓冲溶液浓度,可以改善峰形、提高分离度,实验比较了流动相中不同乙酸铵浓度(10、20、50、100、200 mmol/L)对化合物峰形和信号响应的影响,结果发现,溶血磷脂等部分组分信号响应随乙酸铵浓度升高而降低,而亲水性氨基酸峰形随乙酸铵浓度的增加而变得更好。主要原因有二:1)流动相中乙酸铵的浓度提高,抑制了部分碳水化合物在ESI源的电离,导致信号降低;2)流动相中乙酸铵的浓度加大,增加了色谱柱固定相表面的极性水层的厚度,使得化合物和色谱柱之间的作用更加充分,同时也降低了离子交换作用力的影响,最终峰形更加对称,甚至一些化合物的响应随之增加^[27]^。为了同时兼顾溶血磷脂等组分的信号响应和亲水性氨基酸的峰形,流动相中乙酸铵浓度最终设定为50 mmol/L。

### 2.3 代谢物分析

为了进一步验证所构建的HILIC/RPLC-MS/MS双模式交替分离系统,本研究对14种代表性代谢物进行了分析,MS/MS采集所得提取离子色谱图如图3a所示。为了对比代谢物覆盖度,分别使用RPLC-MS/MS单模式和HILIC-MS/MS单模式对14种代表性代谢物进行了分析,所得提取离子色谱图如图3b和图3c所示。在优化的色谱条件下,使用RPLC-MS/MS单模式方法分析时,两种溶血磷脂不出峰,胆碱峰形较差,且大多数氨基酸在靠近死体积处被洗脱,很可能存在离子抑制。此外,同分异构体肌氨酸和L-丙氨酸未实现充分分离,说明RPLC- MS/MS单模式无法覆盖强极性和强疏水性的代谢物,RPLC-MS/MS单模式只能分析14种代谢物中的6种。使用HILIC-MS/MS单模式方法分析时,所有氨基酸均具有良好的分析物保留性和分离度,包括同分异构体肌氨酸和L-丙氨酸,但是*α*-酮戊二酸和富马酸不出峰,琥珀酸和顺式-乌头酸重复性较差,说明HILIC-MS/MS单模式无法对部分有机酸进行准确定量,HILIC-MS/MS单模式只能分析14种代谢物中的10种。而使用我们所构建的HILIC/RPLC-MS/MS双模式交替方法,可以综合两种单模式分析方法的覆盖度,同时分析14种代谢物,且所有代谢物均有较好的保留,大部分代谢物都能达到基线分离,解决了RPLC-MS/MS单模式和HILIC-MS/MS单模式结合分析时间长的问题。

**图 3:**
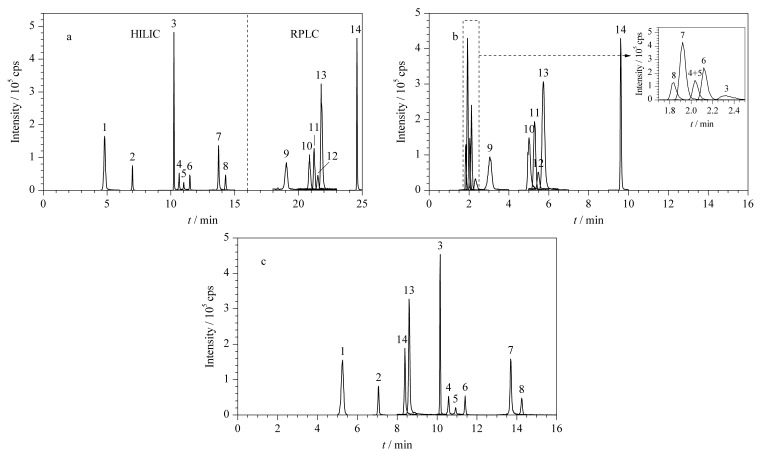
不同分离系统下14种代谢物的提取离子色谱图

### 2.4 方法学评价

选择尿液基质中的14种代表性代谢物对所建立的HILIC/RPLC-MS/MS双模式交替分离方法进行了考察。待测化合物均为内源性物质,在自然界中不存在天然的空白基质,必须选择合适的替代基质用于构建标准曲线和方法验证。本研究依据《中国药典》(2015版)中“生物样品定量分析方法验证指导原则”,采用人工尿样作为空白基质,通过向其中加入不同浓度的混合标准工作液,建立基质匹配标准曲线。以尿液中各化合物的质量浓度为横坐标,化合物峰面积为纵坐标(其中胆碱以化合物峰面积与内标峰面积比值为纵坐标),加权重因子1/*X*进行回归运算得到线性和相关系数(*R*^2^)。根据定量离子信噪比为3(*S/N*=3)和10(*S/N*=10)时对应的样品中待测物浓度,分别得到检出限(LOD)和定量限(LOQ),各目标化合物的线性范围、相关系数及检出限、定量限见表2。结果表明,所有化合物在各自的线性范围内线性关系良好,相关系数均大于0.99,方法的检出限和定量限分别为0.02~42.86 ng/mL和0.08~142.86 ng/mL。

**表 2:** 14种代谢物的线性范围、相关系数、检出限、定量限、回收率及日内、日间精密度

Compound	Linear range/ (ng/mL)		R^2^	LOD/ (ng/mL)	LOQ/ (ng/mL)	Recoveries^*^/% (n=6)	Intra-day RSDs^*^/% (n=6)	Inter-day RSDs^*^/% (n=18)
Sarcosine	10-	2000	0.99758	2.22	7.39	90.5, 92.2, 95.7	5.21, 3.15, 1.94	7.35, 5.68, 4.20
L-Alanine	50-	10000	0.99746	5.18	17.27	106.2, 104.4, 110.5	6.23, 4.29, 3.67	5.80, 4.56, 3.75
L-Histidine	100-	20000	0.99588	1.25	4.16	105.2, 113.9, 112.4	4.62, 2.56, 2.13	6.20, 3.42, 4.13
Lysine	100-	20000	0.99690	3.59	11.96	98.6, 104.2, 105.0	7.22, 4.26, 3.29	7.53, 5.60, 4.46
L-Glutamic acid	50-	10000	0.99833	3.09	10.29	102.7, 108.4, 99.5	5.74, 2.13, 2.55	8.25, 5.32, 4.45
Choline	10-	2000	0.99605	0.02	0.08	93.0, 90.6, 95.5	3.53, 2.15, 1.40	5.42, 4.62, 3.11
16∶0 Lyso PE	1-	200	0.99947	0.16	0.54	102.5, 100.7, 95.6	5.23, 3.56, 1.85	6.30, 4.58, 2.52
18∶0 Lyso PC	1-	200	0.99478	0.06	0.19	99.7, 106.9, 113.2	4.25, 3.26, 0.49	6.62, 4.55, 3.14
L-Leucine	25-	5000	0.99946	1.87	6.23	90.3, 85.2, 89.5	5.46, 4.22, 1.92	4.23, 3.55, 2.40
L-Phenylalanine	25-	5000	0.99997	0.34	1.14	93.2, 96.7, 102.5	3.46, 3.25, 1.03	4.16, 3.72, 2.07
α-Ketoglutaric acid	100-	20000	0.99969	19.35	64.52	85.3, 87.8, 90.4	5.49, 4.32, 1.20	8.70, 5.44, 3.62
cis-Aconitic acid	100-	20000	0.99964	5.34	17.79	97.6, 108.8, 105.7	7.62, 4.36, 1.56	5.22, 3.71, 2.50
Fumaric acid	200-	40000	0.99873	42.86	142.86	96.6, 104.7, 98.0	6.58, 4.22, 1.40	8.20, 5.14, 2.56
Succinic acid	50-	10000	0.99311	4.58	15.27	106.5, 109.1, 112.8	5.20, 3.62, 2.67	7.31, 4.30, 2.82

* Three spiked levels: 5%, 25%, 75% of the highest point of the linear ranges.

分别于空白尿液中加入低、中、高3个水平(线性范围最高点的5%、25%和75%)的混合标准工作液,按优化后的样品前处理办法进行处理,每个水平平行6个样品,测定其回收率和相对标准偏差(RSD)。通过1天内测定3个加标水平下的6个平行样品得到日内精密度,连续3天测定3个加标水平下的6个平行样品得到日间精密度。结果显示,14种代谢物的回收率为85.2%~113.9%,日内及日间精密度RSD范围分别为0.49%~7.62%和2.07%~8.70%,如表2所示,该数据表明新建立的方法准确可靠。

## 3 结论

代谢组学已被广泛应用于疾病相关生物标志物的研究,人们期望获得高覆盖度、高通量和实用性强的方法。本研究在通用液相色谱仪的基础上,增加1个输液泵和2个切换阀,构建了一种基于单进样器的HILIC/RPLC-MS/MS双模式交替分离系统,实现了HILIC和RPLC色谱系统的正交组合,分析通量和覆盖度显著提高。同时,该系统仪器配置简单,自动化程度高,适合大规模的代谢组学研究。
